# Pediatric cirrhotic cardiomyopathy: literature review and effect size estimations of selected parameters

**DOI:** 10.1007/s00431-024-05746-6

**Published:** 2024-09-04

**Authors:** Alexandru-Ștefan Niculae, Simona Sorana Căinap, Alina Grama, Tudor Lucian Pop

**Affiliations:** 2nd Department of Pediatrics, Iuliu Hațieganu University of Medicine and Pharmacy, 3-5 Crișan Street, Cluj-Napoca, Romania

**Keywords:** Liver fibrosis, Cholestasis, Cardiomyopathy, Cirrhosis

## Abstract

Liver cirrhosis is a significant global health concern, and cirrhotic cardiomyopathy (CCM) is a notable complication affecting both adults and children. While CCM is well-studied in adults, understanding its manifestation and diagnostic criteria in pediatric patients remains a challenge. This review explores the evidence for structural and functional cardiac alterations in children with liver cirrhosis. Structural abnormalities, including increased left ventricular mass index (LVMI) and altered left ventricular wall thickness ratios, are prevalent in pediatric CCM. These abnormalities persist even after liver transplantation, highlighting the systemic impact of liver disease. Evidence suggests that altered systolic and diastolic function, as well as electrocardiographic abnormalities such as prolonged QT intervals, are common in pediatric CCM. Blood biomarkers, including brain natriuretic peptide (BNP) and troponin levels, offer insights into cardiac function in pediatric cirrhotic patients. Elevated BNP levels correlate with adverse outcomes, indicating its potential as a prognostic marker. However, further research is needed to elucidate the diagnostic utility of these biomarkers in pediatric CCM. *Conclusion*: This review provides estimates of the standardized mean difference among selected cardiac parameters in children with and without cirrhosis. Tailored diagnostic criteria and comprehensive assessment methods will be essential for accurate diagnosis and effective management of pediatric CCM.

**Table:** 

**What is Known:** • *CCM adds to the burden of care of patients with cirrhosis.* • *Diagnostic criteria for adults are evolving, but there are no specific criteria for pediatric CCM.*
**What is New:** • *Cardiac function in children with cirrhosis indicates some parameters not considered in adults are altered.* • *Effect size estimations for certain parameters provide a guideline for future research into pediatric CCM.*

**What is Known:**

• *CCM adds to the burden of care of patients with cirrhosis.*

• *Diagnostic criteria for adults are evolving, but there are no specific criteria for pediatric CCM.*

**What is New:**

• *Cardiac function in children with cirrhosis indicates some parameters not considered in adults are altered.*

• *Effect size estimations for certain parameters provide a guideline for future research into pediatric CCM.*

## Introduction

Liver cirrhosis accounts for 2.4% of global deaths as per recent estimates [1]. Cirrhotic cardiomyopathy (CCM) is represented by structural, electrophysiologic, and mechanical changes to the heart in patients with liver cirrhosis. Half of patients with cirrhosis fulfill the criteria for CCM, with estimates in adults reaching 70% of those with liver cirrhosis [2–4]. Studies in pediatric patients identified overall lower rates of CCM in children with liver cirrhosis [5, 6], yet children with specific etiologies of cirrhosis, such as that secondary to biliary atresia, have rates of CCM of up to 50% [7].

Criteria for CCM in adult patients have been defined since 2005 [8] by the World Gastroenterology Organization (WGO criteria) and updated recently by the Cirrhotic Cardiomyopathy Consortium (CCC criteria) [9]. Table 1 provides a comprehensive overview of the original and updated proposed criteria for cirrhotic cardiomyopathy in adults. There is considerable debate regarding whether the original WGO criteria or the more recent CCC criteria are superior in adult populations [10].

**Table 1:** Definitions of CCM in 2005 and 2020

	WGO 2005 criteria [9]	CCC 2019 criteria [10]
Systolic dysfunction	• Altered contractility under stress conditions (either pharmacologically induced or various conditions such as fluid overload or physical activity) • LVEF < 55%	• LVEF < 55% • Absolute LVGLS< 18%
Diastolic dysfunction	• DECT > 200 ms • IVRT > 80 ms • *E*/*A* < 1	• Septal *e*′ velocity < 7 cm/s • *E*/*e*′ ≥ 15 • Left atrial volume index > 34 mL/m^2^ • Tricuspid jet velocity > 28 m/s, in the absence of portopulmonary hypertension
Supportive criteria*	• ECG abnormalities: prolonged QTc interval • Abnormal chronotropic response • Electromechanical dissociation • Elevated blood biomarkers such as BNP, proBNP, and troponin • Increased myocardial mass • Dilated left atrium	• ECG abnormalities (no other specifiers) • Abnormal chronotropic or inotropic response • Electromechanical dissociation • Enlargement of heart chambers • Abnormalities on cardiac MRI • Modified myocardial mass

*DECT*, deceleration time; *E/A*, early (*E*) and late (*A*) peak velocity of transmitral valve blood flow with *E*/*A* ratio; *ECG*, electrocardiogram; *E/e′*, transmitral to tissue velocity ratio (*E*/*e*′ ratio); *GLS*, global longitudinal strain; *IVRT*, isovolumetric relaxation time; *LAV(i)*, left atrial volume (index); *LV*, left ventricle; *LVEF*, left ventricle ejection fraction

^*^CCC 2019 additional criteria require additional study

Research into pediatric patients with cardiac dysfunction following liver cirrhosis has often used adult criteria, yet it is unclear whether these criteria perform well in children. Some studies failed to find differences between cirrhotic and non-cirrhotic children for biomarkers that are validated in adults [11]. Other authors have published proposals for specific parameters to be used in pediatric CCM associated with biliary atresia [12].

This review will detail the evidence from pediatric studies concerning CCM criteria as well as the proposal for additional criteria specific to pediatric patients with liver cirrhosis.

## Pathophysiology of CCM

CCM entails functional and structural alterations caused by systemic factors that affect heart function as well as direct changes to cardiac tissue.

Hepatic cirrhosis and subsequent portosystemic shunting lead to vasodilation due to increased signaling by mediators such as nitric oxide (NO) and calcitonin gene-related peptide [8, 13]. This contributes to the hyperdynamic circulation seen in cirrhosis. Subsequently, activation of the renin–angiotensin–aldosterone pathway leads to increasing plasma volume, fluid retention, and development of ascites [4, 8], while vascular resistance stays low.

The cardiac muscle itself changes in cirrhotic patients. Falling mean-arterial blood pressure due to constant relative vasodilation leads to sympathetic activation. Adrenergic stimulation thus induces cardiac hypertrophy, yet, in conjunction with increased inflammation that is characteristic of cirrhosis, the heart muscle also develops fibrosis [3, 4]. Magnetic resonance imaging of the cardiac muscle in adults with cirrhosis revealed increased myocardial fibrosis and the severity of fibrosis has been correlated to serum markers of inflammation [14]. Thus, altered systolic function, especially under stress conditions, is prominent. Circulating NO, altered beta-adrenergic signaling in cardiomyocytes, and altered calcium homeostasis also contribute to poor systolic function in CCM [3].

Cardiac muscle hypertrophy and fibrosis reduce the compliance of the ventricular walls during diastole, thus contributing to altered diastolic function in CCM [8, 13]. This causes abnormal ventricular filling patterns during the cardiac cycles, with increased end-diastolic ventricular pressures, limitation of blood flow through the mitral valve, and rising pressures in the left atrium [4].

Finally, electrocardiographic (ECG) abnormalities are also associated with CCM, the most prominent of which is prolongation of the rate-corrected QT interval (QTc). High blood levels of bile acids have been considered a causative factor for ECG abnormalities, and altered regulation of the plasma membrane potassium channel has been cited as a factor in QTc prolongation in animal models [3]. Data gathered from patients with cirrhosis indicated that prolonged QT is related to increased myocardial mass [15], with both parameters showing a trend toward normalization after liver transplant (LT) surgery.

## Cardiac structural abnormalities in pediatric CCM

Left atrial volume index (LAVI, indexed to height-squared) is the only structural parameter included in the evaluation of adult CCM as part of the newly proposed 2019 criteria [9]. However, in pediatric studies, several structural abnormalities were identified.

A retrospective analysis of pediatric candidates for LT, published in 2002, showed significantly larger systolic left ventricular posterior wall thickness (LVPWTs) in this group compared to healthy sex- and age-matched controls [16].

Left ventricular mass and left ventricular mass index (LVMI) have been identified as abnormal in pediatric patients with liver cirrhosis. Celtik et al. published evidence [6] that LVMI (calculated as grams/body surface area) is higher in children with cirrhosis than those with liver disease that is not as advanced. Both groups individually show higher LVMI compared to healthy controls.

Similar findings come from studies that have observed structural cardiac parameters before and after LT. LVMI decreased significantly after LT. Relative wall thickness (RWT) and left ventricular internal diameter during diastole (LVIDd) were larger in patients before they had their transplant [17]. One caveat is that the LVMI was calculated as an index of left ventricular mass divided by the height raised to the power of 2.7, a method considered to render better results of LVM corrected for anthropometric variations, age, and gender [18, 19].

One of the largest retrospective studies of pediatric LT recipients evaluated 198 patients’ echocardiographic measurements before and after LT. Patients were categorized as “advanced fibrosis” (aF, with an ISHAK fibrosis score of 0–3) or “non-advanced fibrosis” (naF, with an ISHAK fibrosis score 4–6). Echocardiographic evaluations of these patients before LT revealed higher LVM, LVMI, and LVIDd in the aF group compared to the naF group. At a second echocardiographic evaluation, 1 year after LT, patients showed lower values of LVIDd, LVM, and LVMI compared to their measurements before LT. Also, pre-LT LVM and LVMI were significantly correlated to total blood bilirubin values, suggesting that cholestasis plays a prominent role in these features of CCM [11].

More evidence concerning pediatric CCM comes from studies concerning biliary atresia (BA). BA involves the rapid obliteration of bile ducts with subsequent rapid degradation of liver functions and cirrhosis [20]. Children require prompt diagnosis and surgical intervention (the Kasai portoenterostomy procedure, KPE) [21]. Delayed recognition of this disease leads to the rapid development of cirrhosis and the requirement for LT. Children with cirrhosis due to BA show similar structural heart abnormalities as those reported in cohorts of patients with diverse etiologies of cirrhosis.

The first large study of CCM in children with BA-associated cirrhosis was published in 2011 and involved 40 children with BA who had LT. These patients showed higher LVM, LVMI (indexed both to body surface area and height^2.7^), IVS thickness, and LV free wall thickness compared to controls. Moreover, cardiac structural alterations observed pre-LT were correlated with the length of stay in the intensive-care unit after LT. Interestingly, the authors did not find any children with congenital heart disease among their cohort of BA patients [7]. A more recent publication from the same group, involving a new group of patients with BA and requiring LT [12], evaluated structural heart parameters in 69 pediatric LT candidates aiming to validate specific criteria for pediatric CCM associated with BA. The authors reported that a LVMI > 95 g/m^2.7^ or a RWT > 0.42 were good candidates for defining CCM associated with BA since these cut-off values performed well in identifying patients that had higher rates of multi-organ dysfunction before or after LT, serious adverse outcomes of LT, longer ICU length-of-stay after LT, or a higher risk of death both before or after the LT.

Further evidence has been published recently, concerning the relation of CCM in children with BA and the outcomes of the KPE surgical intervention [22]. Children who had higher LVMI went on to have higher rates of non-draining KPE (compared with those with successful KPE or those without KPE attempt). It is important to note that cirrhosis was not a feature of the children included in this study, yet high rates of LVMI above the 95th percentile compared to normal values published were found in these children [23]. Thus, structural cardiac alterations are a feature of BA early in the course of the disease.

Echocardiographic results from children with BA who underwent LT (at a median age of 7.8 months) showed similar results regarding LVMI and RWT in relation to adverse outcomes. Patients with serious adverse events (SAE) after LT turned out to have had higher LVMI and RWT at their pre-LT echocardiographic evaluation. Moreover, AUROC analyses indicated that LVMI > 68 g/m^2.7^ or RWT > 0.41 are good predictors of death or SAE after LT [24]. In another study, the authors found that the diastolic diameter of the right ventricle (RVDd) is increased in pre-LT children compared to controls. Also LVMI, RWT, and measurements of parameters used to calculate LVMI and RWT (namely IVS, LVPWT, and LVDd) are significantly increased in children with BA post-LT, compared to healthy controls [25].

Finally, there is evidence of structural cardiac abnormalities consistent with the abnormalities found in CCM but from children with non-cirrhotic chronic hepatitis. A study of pediatric participants with autoimmune hepatitis revealed greater values of the measurements of the IVS and LVPW compared to healthy controls [26]. In summary, a common feature identified by many studies of pediatric CCM is that the mass of the left ventricle is higher in CCM. Thus, LVMI, either indexed to body surface area or height^2.7^, and  RWT of the left ventricle can be helpful markers for further study and validation. It is important to note that LVM and  RWT are not directly measured. They are calculated using specific measurements of the interventricular septum (IVS), left ventricular posterior wall (LVPW), and left ventricular internal diameter (LVID), taken both in systole and diastole [17].

An interesting addition to the relation between liver dysfunction and cardiomyopathy can be observed from the scientific literature regarding children with Fontan procedure (FP) who subsequently developed Fontan-associated liver disease (FALD) [27, 28]. Structural as well as functional cardiac abnormalities have been identified in patients with FALD and some of these measurements are correlated with the degree of liver stiffness.

One study found that in children and adolescents after FP, liver stiffness is proportional to the time since the FP, but no correlation of the liver stiffness measurements with pulmonary vascular resistance was found [29]. Another study found that after FP, the cardiac index is inversely correlated with bilirubin levels and other scores of hepatic dysfunction [30].

However, we note that drawing conclusions regarding the influence of FALD on cardiac function is complicated by the fact that cardiac abnormalities predate FALD by definition, since these patients have congenital heart disease. This issue could be further clarified by observations of patients who receive heart transplant (HT) alone, by monitoring cardiac parameters of the transplanted heart in relation to the preexistent liver fibrosis.

There is a caveat in relation to the data that suggests that cardiac parameters improve after LT. The cited studies make no mention of the immunosuppressive medication used by patients who have undergone LT. However, tacrolimus is often used for immunosuppression after LT and this medication has been linked to hypertrophic changes of the myocardium, in both adults and children [31, 32]. One of the earliest studies that have comprehensively evaluated structural echocardiographic findings in children receiving tacrolimus after LT identified a subset of the participants that exhibited hypertrophic changes of the myocardium [33]. This complicates the notion that after successful LT, improvement of cardiac parameters is to be expected and underscores the need for a better approach to monitoring heart function in patients, particularly those who use tacrolimus.

## Evidence of systolic dysfunction in pediatric CCM

Altered systolic function is a marker of CCM in adults and indicates inadequate adaptation of cardiac output to increased fluid load. This can be measured as left ventricular ejection fraction (LVEF) or as absolute strain values [34].

As with structural parameters, systolic function parameters have been evaluated in children with liver cirrhosis, yet the results are not as consistent as those regarding LVMI or RWT. In some studies, no differences were found in LVEF or LV fractional shortening (LVFS) in patients with biopsy-proven cirrhosis compared to controls [16]. Using the 2005 WGO criteria, Jang et al. did not find systolic dysfunction in children with LT for diverse liver diseases [5].

However, Celtik and colleagues found significantly higher LVEF and LVFS in cirrhotic patients compared to healthy controls. There was no such difference between patients with portal hypertension who did not have cirrhosis and controls [6].

In a cohort of 40 children with BA, described in 2011 [7], LVFS was greater in BA patients compared to controls. In other studies, LVEF and LVFS did not differ between patients with BA with SAE after LT compared to those without SAE [24] or between patients with BA with successful KPE versus a failed KPE [22].

Global longitudinal strain (GLS) values of the LV (LVGLS) are considered better markers of systolic function in adults compared to LVEF [34, 35]. They have been used to describe changes consistent with CCM in adults [34, 36, 37]. Different research groups arrived at conflicting results regarding GLS values in association with the severity of CLD and prognosis. Thus, caution is still advised when using this parameter to define systolic dysfunction as criteria for CCM [38].

Absolute GLS values were lower in children awaiting LT and after LT, compared to their respective controls [25]. The authors also found that approximately one-third of patients, both before and after LT, show lower LVGLS values compared to normative data previously published in a meta-analysis [39]. The same study indicated that LVEF is also higher in children awaiting LT.

However, to date, despite the availability of published normal values of GLS for different age intervals [40], no results evaluating pediatric CCM via myocardial strain or myocardial work measurements have been published.

## Evidence of diastolic dysfunction in pediatric CCM

Diastolic dysfunction is another well-described feature of CCM in adults, and this is the area where criteria have evolved the most between the previous 2005 WGO criteria and the revised CCC criteria (see Table 1).

In a study that included 45 children awaiting LT, 13% were evaluated for features of LV diastolic dysfunction (LVDD) [5] as defined by the WGO criteria. Children with a Child-Turcotte-Pugh score of 10 or larger had significantly lower ratios of early-to-late mitral valve inflow velocities (*E*/*A* ratio). Moreover, children with LVDD had a significantly longer stay in hospital following their LT procedure compared to cirrhotic children without features of LVDD.

Mitral-valve inflow (*E*/*e*′ ratio) was not different before and 6 months after LT [17]. Patients with portal hypertension and cirrhosis had longer *E* wave duration, lower deceleration times (DECT), and myocardial *A* velocities (Am) compared to controls. However, the authors of this study used modified CCM criteria based on the 2005 WGO definitions to define a group of ten patients with CCM out of a total of 40 with cirrhotic portal hypertension. When this group of patients was compared to the healthy control group, the authors found significantly longer DECT and *E*/*A* ratios compared to controls.

In a retrospective cohort of children with BA, diastolic function was evaluated by the *E*/*A* ratio, which was not different between the SAE groups and the no SAE group [24].

In a study of patients with chronic hepatitis B virus (HBV) infection, those with more severe hepatitis showed parameters suggestive of diastolic dysfunction of the right ventricle: longer isovolumetric relaxation times (IVRT), decreased peak early myocardial tissue velocity (*E*′), and decreased peak early myocardial tissue velocity-to-peak late myocardial tissue velocity (*E*′/*A*′), compared to controls [41]. In patients with autoimmune liver disease, conventional 2D echocardiographic measurements (*E* wave, *A* wave, and *E*/*A* ratios measured at mitral and tricuspid valves) show no difference compared to controls. However, using Doppler imaging techniques, these authors report significant dysfunction in systolic and diastolic function parameters in both ventricles [26].

## Abnormalities on electrocardiogram in pediatric CCM

The rate-corrected QT (QTc) interval is an important feature of CCM, yet few studies concerning children with chronic liver disease and cirrhosis have investigated this parameter.

The earliest evidence concerning children was published in 1999, measuring QTc interval in 38 children with acute liver failure awaiting LT, but only 3 of them were diagnosed with cirrhosis. Seven children in this group showed a prolonged QTc interval, defined as a rate-corrected QT interval above 450 ms. Those who survived until after LT showed normalization of their QTc values. No significant differences were found on standard liver function tests between patients with prolonged QTc and those with normal QTc in this study [42].

A study of 88 children with chronic liver disease (CLD) of diverse etiologies evaluated the relationship between QTc, LT outcomes, and QTc after LT [43]. A prolonged QTc interval was found in almost half of the CLD patients in this study. The mean QTc interval was longer compared to healthy controls. Those with prolonged QTc intervals had meaningfully higher PELD scores and had a greater risk of death compared to children with CLD.

Echocardiographic parameters measured before surgery in children with CLD undergoing LT indicated that an LVMI equal to or greater than 82.51 g/m^2^ before LT led to increased odds of QTc prolongation. Half of participants showed prolonged QTc > 440 ms before LT and one-third of the children had QTc > 450 ms [15].

QTc was shown to be larger in children with cirrhotic portal hypertension compared to healthy controls, as well as in children with portal hypertension but who have not yet developed cirrhosis [6].

However, Junge et al., when comparing the QTc interval among patients with advanced versus non-advanced liver fibrosis, found no statistically significant difference between the two groups [11]. An estimation of the effect size of cirrhosis on QTc values derived from published values is provided in Table 2.

**Table 2:** Effect sizes of selected structural and functional cardiac parameters. All results were reported as significant with null-hypothesis inference testing in the original reports

Cardiac parameter	Study	Patients vs controls (number of participants in each group)	Hedge’s *g*	Glass’s delta^Ɨ^
QTc interval	Arikan et al., 2008 [42]	88 pre-LT children vs healthy controls (66)	0.29	1
	Celtik et al., 2015 [7]	Cirrhotic PHT (40) vs healthy controls (50)	1.14	1.33
LVGLS	Brito et al., 2021 [29]	BA-associated cirrhosis (30) vs healthy controls (30)	0.89	1.27
LVMI	Brito et al., 2021 LVM indexed to height^2.7^, measured in meters	BA-associated cirrhosis (30) vs healthy controls (30)	1.88	4.83
	Desai et al., 2011 [8] LVM indexed to BSA (m^2^)	Pre-LT surgery BA patients (40) vs healthy controls (30)	1.33	2.82
	Celtik et al., 2015 [7] LVM indexed to BSA (m^2^)	Cirrhotic PHT (40) vs healthy controls (50)	0.72	0.86
DECT	Brito et al., 2021 [29]	BA-associated cirrhosis (30) vs healthy controls (30)	0.67	0.85
	Celtik et al., 2015 [7]	Cirrhotic PHT (40) vs healthy controls (50)	− 0.07^¶^	0.09^¤^ (raw value)
IVRT (measured at the lateral wall of the LV)	Brito et al., 2021 [29]	BA-associated cirrhosis (30) vs healthy controls (30)	0.61	0.86

^¶^Negative value for Hedge’s *g* and indicates that in this study, patients had lower QTc compared to controls

^¤^Raw computed value for Glass’s delta for DECT in this study is positive, indicating that controls had higher DECT than patients; result in accordance with ^¶^

^Ɨ^All values for Glass’s delta are presented as absolute values; computationally, Glass’s delta yields negative values when patients have higher mean values compared to controls

## Blood biomarkers of cardiac function in pediatric CCM

The earliest results reporting on plasma biomarkers of cardiac function were published by Shirakami et al., involving 18 patients who underwent LT for BA-associated cirrhosis. Plasma levels of brain natriuretic peptide (BNP), as well as other markers of cardiovascular function, were elevated 30 min prior to the start of LT surgery [44] in these patients compared to normal values published in the literature at the time.

A study of BNP and endothelin (ET) plasma levels in pediatric LT recipients several years after LT revealed no significant differences compared to healthy controls and no correlation of these peptides with echocardiographic structural and functional parameters [45].

More recent studies have also investigated blood values of BNP in children with cirrhosis. Fattouh and colleagues found higher levels of serum BNP in 52 children with cirrhosis of different etiologies compared to healthy controls. In the same study, some echocardiographic parameters were also significantly different in patients, such as higher LVPWT and lower *E*/*A* ratios in patients — in accordance with other studies. However, there was a significant correlation between echocardiographic parameters and BNP levels in these patients [46].

In patients with BA with successful KPE, BNP levels were positively correlated with the Child-Turcotte-Pugh score and the APRI and FIB-4 scores. Patients with splenomegaly or with esophageal varices also had significantly higher BNP levels compared to children with BA without these features.

Finally, in the Brazilian cohort, BNP levels are inversely correlated with absolute values of LVGLS, meaning that lower levels of BNP indicate better LV contractility [25].

Less evidence has been published concerning troponin levels in children with cirrhosis. Levels of serum troponin recorded intra-operatively during LT surgery were negatively correlated with pre-LT albumin levels and positively correlated with pre-LT bilirubin levels, respectively, in a retrospective study of 123 children with BA that required LT [47]. This suggests that the amount of troponin released from the myocardium during LT surgery is partly due to the magnitude of the native liver dysfunction before surgery.

## Estimation of effect sizes of selected echocardiographic parameters comparing children with cirrhosis with healthy controls

Table 2 presents effect size values for selected parameters (calculated as Hedge’s *g*, an unbiased version of Cohen’s *d*, and Glass’s delta) [48, 49]. These parameters were identified as abnormal in cirrhotic children and are also part of the criteria proposed for the evaluation of CCM in adults (see Table 1). Comparisons to healthy controls have not been published very often [6, 25]. Yet, some or all of these parameters are significantly different even compared to children with other CLD without cirrhosis [6, 11] or have been associated with risks of adverse outcomes during disease progression or following LT surgery [12, 24].

Calculated effect sizes for QTc, LVMI, and LVGLS differences between children with cirrhosis and healthy controls indicate significant differences, even when using a pooled standard deviation as a unit of measure. These results support published conclusions that these parameters are clinically relevant, leading to poorer outcomes for children during disease progression and in relation to LT surgery and survival [12, 24]. Additionally, the large effect sizes indicate further efforts to formally describe pediatric CCM should focus on these parameters.

Close cardiac monitoring of patients with CLD could provide important early warning signs of CCM. As shown by Celtik et al. [6], patients can present with modified cardiac function parameters well before cirrhosis sets in.

As demonstrated by Li et al., cardiac parameters have a good discriminating ability with regard to serious adverse events after LT surgery [24]. Given the rapid progression of BA to cirrhosis, this group of patients provides an opportunity to monitor and characterize cardiac function closely as the liver disease progresses.

## Perspectives for future research

In researching the topic of pediatric CCM, it is important to note that pediatric guidelines or recommendations on diagnosing and treating this condition are lacking, and the clinical approach is most often extrapolated from experience with adult patients. In addition to pediatric CCM, other complications related to end-stage liver disease, such as hepato-renal syndrome or hepato-pulmonary syndrome, similarly lack formal guidelines in pediatric practice. For example, although data from adult studies indicate that there is no causal relationship between CCM and hepato-pulmonary syndrome [50], providing independent data from pediatric participants with these disorders must be a priority.

Studying pediatric CCM in the near future should focus on three directions. First, diagnostic criteria appropriate for pediatric patients with CCM must be established. These should include a thorough evaluation of echocardiographic, electrocardiographic, and serum parameters, starting with those described in this paper. Large tertiary centers where children receive LT and post-LT care are well placed to initially conduct retrospective studies. Second, validating these biomarkers can be done both retrospectively and prospectively in carefully curated cohorts of pediatric patients. GLS measurements could provide a novel echocardiographic approach to describing cardiomyopathy in these patients. However, there are challenges associated with these procedures in children: longer examination times, the need for additional expertise, and reduced cooperation from children for this procedure. Last, focusing on detecting early changes associated with CCM as fibrosis progresses, but before cirrhosis develops, is key. Quantitative studies to determine effect sizes (correlation coefficients, odds ratios, mean differences) in patients with earlier stages of fibrosis is key. This can be done in parallel with developing screening programs for CCM (as well as other end organ dysfunctions) in at-risk patient groups who are known to have liver disease but have yet to develop cirrhosis.

With improved detection of CLD and survival of children after LT, there will be a clear need for an organized approach to cardiac monitoring in these patients, especially those that presented clear features of CCM before LT [51].

No formal recommendations for cardiac follow-up of children with CLD exist. Data from adult patients suggest follow-up after LT at 1, 6, and 24 months post-LT [10, 34, 52]. However, given that results from pediatric cohorts indicate cardiac dysfunction precedes and worsens with the development of cirrhosis, a more proactive assessment schedule might provide significant benefits for patients. At a minimum, monitoring the development of progressive liver fibrosis and including patients with more advanced fibrosis in a cardiac screening schedule could be appropriate and cost-effective. It is worth noting that studies evaluating the feasibility, usefulness, and cost-effectiveness of such an approach have not been conducted.

## Conclusions

Pediatric CCM remains an important part of understanding the needs and risks of children with liver cirrhosis. This review has summarized the evidence concerning the main structural and functional parameters that characterize this condition. Using modern methods to quantify systolic functions in children with cirrhosis, such as GLS or pressure-strain loop-derived measurements of myocardial work, could provide more robust descriptions of pediatric CCM. Effect sizes derived from results published in the mentioned studies provide a strong indication that the parameters summarized in this review are viable candidates for inclusion in formal diagnostic criteria of pediatric CCM.

## Data Availability

No datasets were generated or analyzed during the current study.
